# Expression and significance of INTS10 and IRF3 in chronic hepatitis B patients

**DOI:** 10.3389/fmicb.2025.1609297

**Published:** 2025-11-26

**Authors:** Aizhu Ye, Xiao Chen, Dongbiao Qiu, Wennan Wu

**Affiliations:** 1 Department of Blood Transfusion, The First Affiliated Hospital of Fujian Medical University, Fuzhou, China; 2 Department of Blood Transfusion, National Regional Medical Center, Binhai Campus of the First Affiliated Hospital, Fujian Medical University, Fuzhou, China; 3 Department of Laboratory Medicine, Fujian Key Laboratory of Laboratory Medicine, Gene Diagnosis Research Center, Fujian Clinical Research Center for Clinical Immunology Laboratory Test, The First Affiliated Hospital, Fujian Medical University, Fuzhou, China; 4 Department of Laboratory Medicine, National Regional Medical Center, Binhai Campus of the First Affiliated Hospital, Fujian Medical University, Fuzhou, China

**Keywords:** INTS10, hepatitis B virus (HBV), chronic hepatitis B (CHB), interferon regulatory factor 3 (IRF3), HBV infection

## Abstract

**Introduction:**

Hepatitis B virus (HBV) infection continues to pose a significant global health challenge, affecting millions of individuals worldwide. Persistent HBV infection is acknowledged as a complex and polygenic process influenced by virological, environmental, and host genetic factors. Previous in vitro studies have indicated that INTS10 inhibits HBV replication in a manner dependent on interferon regulatory factor 3 (IRF3). Nevertheless, the relationships among INTS10 expression, IRF3, and HBV replication in patients with chronic hepatitis B (CHB) have not been comprehensively examined. Thus, this study aimed to investigate the associations between INTS10, IRF3, and HBV replication in CHB patients.

**Methods:**

A total of 127 treatment-naïve CHB patients were recruited and categorized into hepatitis B e antigen (HBeAg)-negative and HBeAg-positive groups. Quantitative PCR was utilized to quantify INTS10 and IRF3 expression levels, alongside serum HBV DNA load and conventional liver biochemical parameters, to evaluate HBV infection status.

**Results:**

Our findings revealed significantly lower expression levels of both INTS10 and IRF3 in HBeAg-positive patients compared to their HBeAg-negative counterparts. Additionally, a positive correlation was observed between IRF3 and INTS10 expression. In HBeAg-positive CHB patients, INTS10 and IRF3 levels exhibited negative correlations with HBV DNA, HBeAg, hepatitis B surface antigen (HBsAg), and total bile acid (TBA) levels. Conversely, no significant correlations were detected between INTS10 or IRF3 and these virological and biochemical markers in HBeAg-negative patients.

**Discussion:**

These results suggest that the relationship between INTS10 and IRF3 expression may be modulated by HBeAg status. This implies a potential involvement of HBeAg in the antiviral mechanisms mediated by INTS10 and IRF3 in CHB. Consequently, our study offers valuable insights that may contribute to enhanced clinical management of HBV infection.

## 1 Introduction

HBV infection represents a major global health burden, affecting more than 250 million individuals worldwide and leading to a spectrum of clinical outcomes, including CHB, cirrhosis, and hepatocellular carcinoma (HCC) (Cornberg et al., 2025; Ye et al., 2024). The progression to chronic infection or spontaneous viral clearance is recognized as a multifactorial process shaped by virological, environmental, and host genetic factors (Lin et al., 2025). Despite advances in understanding these determinants, the precise molecular mechanisms underlying CHB persistence remain incompletely elucidated, posing ongoing clinical challenges and necessitating further investigation.

INTS10 is a component of the integrator complex, a multi-subunit assembly involved in transcriptional regulation (Mendoza-Figueroa et al., 2020). A recent genome-wide association study (GWAS) conducted in a Chinese population identified a novel susceptibility locus near the INTS10 gene, where the protective allele was associated with elevated hepatic INTS10 expression. Functional follow-up studies demonstrated that INTS10 suppresses HBV replication in hepatocyte cell lines through a mechanism dependent on IRF3 (Li et al., 2016).

IRF3, a key member of the interferon regulatory factor family, plays a central role in innate antiviral immune responses by regulating the expression of interferon-stimulated genes (Negishi et al., 2018). Previous work has shown that both INTS10 and IRF3 expression are reduced in patients with chronic hepatitis B compared to individuals who have achieved spontaneous viral resolution (Li et al., 2016). However, the relationship between INTS10 and IRF3 expression and their collective impact on HBV replication in CHB patients remains poorly defined. Therefore, this study aimed to clarify the interplay among INTS10, IRF3, and HBV replication in a well-characterized cohort of CHB patients.

## 2 Materials and methods

### 2.1 Subjects

During the period from July 2018 to March 2022, a total of 127 treatment-naïve Chinese Han patients with CHB, aged 30–45 years, were enrolled in this study. These patients were selected after excluding most other known causes of liver disease. The exclusion criteria are listed below: (1) Age < 18 years old; (2) Co-infected with HCV, HDV, or HIV; (3) Immunocompromised individuals; (4) Patients with concurrent other types of malignancies/tumors; (5) Patients with liver failure; (6) Patients with other autoimmune diseases. Based on their HBeAg status, the participants were categorized into two groups: 61 were HBeAg-positive and 66 were HBeAg-negative. The diagnosis of CHB was established in accordance with the 2025 Clinical Practice Guidelines on the management of hepatitis B virus infection (EASL) (Cornberg et al., 2025). All subjects were of Chinese Han ethnicity and genetically unrelated. The study protocol was approved by the Ethics Committee of the First Affiliated Hospital of Fujian Medical University, and written informed consent was obtained from each participant prior to enrollment.

### 2.2 Serological indicators and HBV DNA assays

Biochemical indicator were identified through an automated biochemical method (Roche, Switzerland). The quantification of serum HBV DNA was detected by using ABI 7500 Real-Time PCR System (Life Technologies, USA). Serum hepatitis B surface antigen (HBsAg), HBeAg and hepatitis B core antigen (HBcAg) were detected using the ARCHITECT i2000SR automatic biochemical immunoassay system (Abbott Laboratories, USA). AST, ALT and TBA were determined using an automated biochemical technique (Roche Diagnostics Switzerland) according to the manufacturer’s instructions.

### 2.3 qPCR analysis

Purified RNA was reversely transcribed into cDNA using the RevertAid First Strand cDNA Synthesis Kit (AG, China). qPCR analysis was performed with ABI 7500 Real-Time PCR System (Life Technologies, USA) using TB Green® Premix Ex Taq™ (AG, China). The thermal profile was as follows: pre-denaturation at 95 °C for 30s), 40 cycles of denaturation at 95 °C for 5 s, 62 °C for 30s followed by melt curve analysis. The primer pairs used to detect INTS10 and IRF3 were as follows: 5′- GTCCTAATGCCCCGAGCCAA −3′ and 5′ - GCCGAACAGTCTTCATCGTCT - 3′ for INTS10, 5’-GGCCCCACTCCCAGATCCG −3′ and 5’-GGCTGTCACCTCGAACTCCC-3′ for IRF3, 5′ -CACATGGCCTCCAAGGAGTAAG- 3′ and 5’-TGAGGGTCTCTCTCTTCCTCTTGT- 3′ for GAPDH, respectively.

### 2.4 Statistical analyses

Continuous variables were analyzed using the Student’s *t*-test, while categorical variables were evaluated with the χ^2^ test. Correlations were assessed using Spearman’s correlation test. The fold changes in mRNA expression levels of INTS10 and IRF3 between the HBeAg-positive and HBeAg-negative groups were calculated via the 2^(-ΔΔCT)^ method. All statistical analyses were performed with SPSS version 20.0 (SPSS, Inc., USA). A two-tailed significance level was adopted for all tests, and a *p*-value of less than 0.05 was considered statistically significant. Potential outliers were identified using the box plot method, defined as data points deviating from the group mean by more than ±3 standard deviations.

## 3 Result

### 3.1 Characteristics of the study subjects

The baseline characteristics of the study participants are summarized in Table 1. The enrolled individuals were aged between 30 and 45 years. Among the 61 HBeAg-positive patients, 36 (59.0%) were male and 25 (41.0%) were female, with a mean age of 37.02 ± 4.75 years. The HBeAg-negative patients showed no significant difference in age, with a mean of 39.57 ± 4.11 years (*p* = 0.428). Similarly, no significant difference in gender distribution was observed between the HBeAg-negative and HBeAg-positive groups (*p* = 0.069). In addition, significant differences between the groups were observed in the concentrations of HBsAg and HBeAg, as well as the serum levels of ALT, AST, and TBA (all *p* < 0.05, Table 1). However, no significant differences among the groups were observed in the concentrations of HBcAg and HBV DNA (all *p* > 0.05, Table 1).

**Table 1:** General characteristics of the study population.

Demographic characteristics	HBeAg-negative	HBeAg-positive	*P*
Age	39.57 ± 4.11	37.02 ± 4.75	0.428
Gender			
Male	52 (78.8%)	36 (59%)	
Female	14 (21.2%)	25 (41%)	0.069
HBV infection indicators			
HBsAg (IU/mL)	2030.15 ± 2493.78	10089.16 ± 9136.90	**<0.001**
HBeAg (S/CO)	0.37 ± 0.23	748.87 ± 539.12	**<0.001**
HBcAg (S/CO)	10.72 ± 3.26	10.44 ± 2.73	0.636
HBV DNA (lgIU/ml)	/	7.23 ± 1.23	/
ALT (U/L)	28.27 ± 10.74	228.31 ± 341.56	**<0.001**
AST (U/L)	24.01 ± 13.29	141.47 ± 245.29	**<0.001**
TBA (umol/mL)	5.89 ± 4.88	12.21 ± 6.20	**<0.001**

Measurement data were expressed as median ± SD. Enumeration data were presented as number (%) for every group. ALT, alanine aminotransferase; AST, aspartate aminotransferase; TBA, total bile acid. Bold values mean *p < 0.05, **p < 0.01, ***p < 0.001.

### 3.2 Compared mRNA expression of INTS10 and IRF3 in HBeAg-positive and HBeAg-negative CHB patients

**Figure 1:**
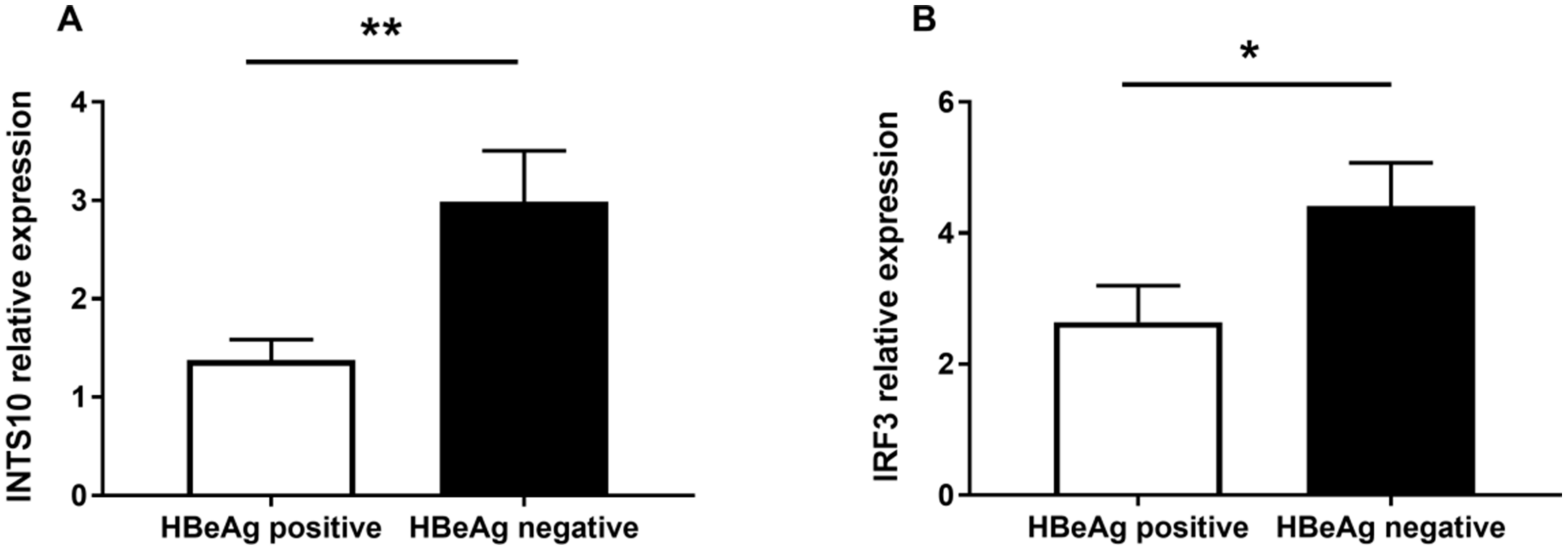
Real-time PCR analysis of mRNA Levels of INTS10 and IRF3 in Positive-HBeAg patients and Negative-HBeAg patients. **(A)** INTS10 mRNA levels in patients with positive-HBeAg was markedly lower than that in the negative HBeAg patients (*p* = 0.006). **(B)** IRF3 mRNA were slightly lower in positive HBeAg patients than those negative patients (*p* = 0.044). The two-tailed *p* values were evaluated by Students’ *t*-test. *p*-values were considered to be significant when below 0.05.

To evaluate the expression levels of INTS10 in HBeAg-positive and HBeAg-negative groups, we quantified INTS10 mRNA levels using real-time fluorescence quantitative PCR in all 61 HBeAg-positive and 66 HBeAg-negative patients. As summarized in Table 2, both INTS10 and IRF3 mRNA expression differed significantly between the two groups. Specifically, INTS10 mRNA levels were markedly higher in HBeAg-negative patients than in HBeAg-positive patients (*p* = 0.006, Figure 1A).

**Table 2:** Relative expression of INTS10 mRNA and IRF3 mRNA in HBeAg-negative and HBeAg-negative groups.

Variable	HBeAg-positive	HBeAg-negative	*P*
INTS10	1.37 ± 1.64	2.99 ± 4.22	**0.006**
IRF3	2.64 ± 4.28	4.41 ± 5.26	**0.044**

Measurement data were expressed as median ± SD. Bold values mean *p < 0.05, **p < 0.01, ***p < 0.001.

Given that IRF3 is a key component of the retinoic acid-inducible gene I (RIG-I) signaling pathway and previous evidence suggests that INTS10 may exert its antiviral effects by upregulating IRF3—thereby enhancing type III interferon production and suppressing HBV replication—we further examined IRF3 expression across all subjects. Consistent with the INTS10 results, IRF3 mRNA expression was significantly elevated in the HBeAg-negative group compared to the HBeAg-positive group (*p* = 0.044, Figure 1B).

### 3.3 Correlation between INTS10 and IRF3 in CHB patients

**Figure 2:**
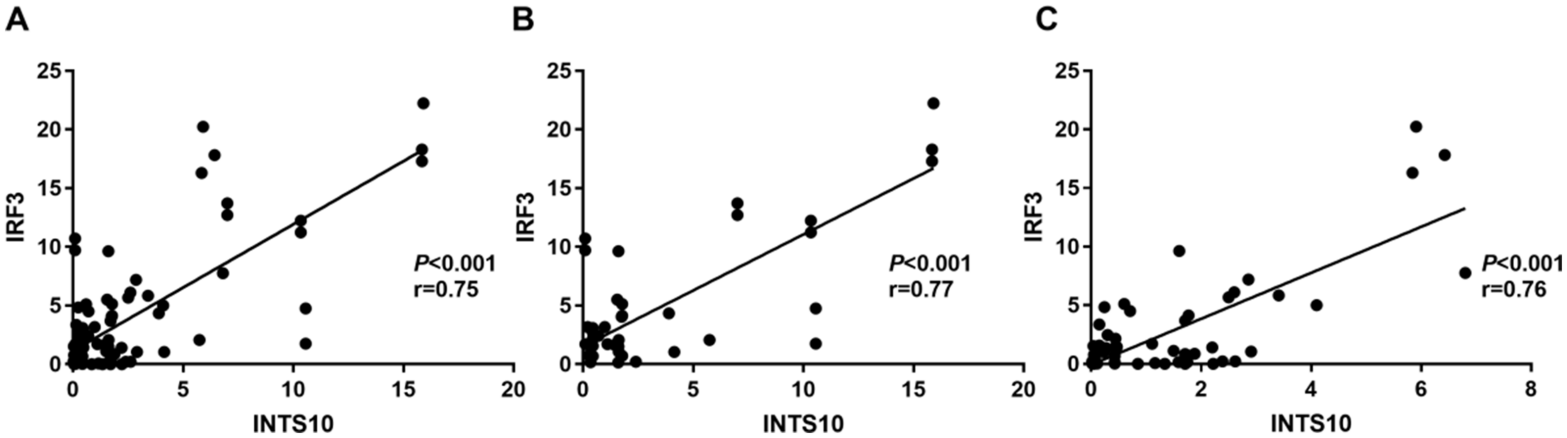
Correlation between INTS10 and IRF3 in CHB patients. **(A)** Correlation between INTS10 mRNA and IRF3 mRNA in all subjects; **(B)** correlation between INTS10 mRNA and IRF3 mRNA in HBeAg negative group; **(C)** correlation between INTS10 mRNA and IRF3 mRNA in HBeAg positive group.

Linear correlation analysis revealed a strong positive association between INTS10 and IRF3 mRNA expression across all CHB patients (r = 0.75, *p* < 0.0001; Figure 2A). This correlation remained consistently significant when analyzed separately in both the HBeAg-negative (r = 0.77, *p* < 0.0001; Figure 2B) and HBeAg-positive (r = 0.76, *p* < 0.0001; Figure 2C) groups.

### 3.4 Associations of INTS10 and IRF3 with HBV infection indicators in HBeAg-positive patients

**Figure 3:**
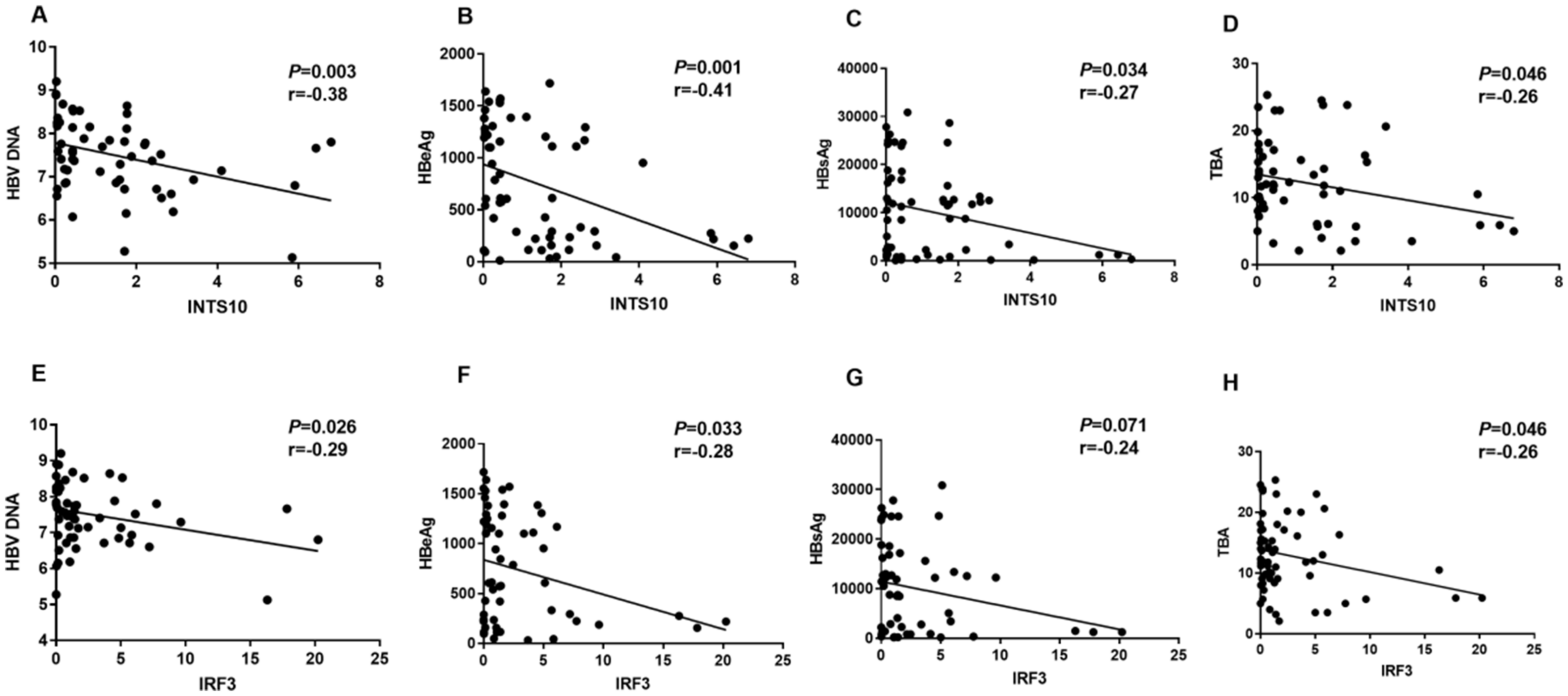
Correlation between the levels of INTS10, IRF3, HBV DNA, HBeAg, HBsAg and TBA in HBeAg positive group. **(A)** Correlation between the INTS10 mRNA and HBV virus load in positive HBeAg group, HBV DNA load were log10 transformed. **(B)** Correlation between the INTS10 mRNA and HBeAg in positive HBeAg group. **(C)** Correlation between the INTS10 mRNA and HBsAg in positive HBeAg group. **(D)** Correlation between the INTS10 mRNA and TBA in positive HBeAg group. **(E)** Correlation between the IRF3 mRNA and HBV virus load in positive HBeAg group, HBV DNA load were log10 transformed. **(F)** Correlation between the IRF3 mRNA and HBeAg in positive HBeAg group. **(G)** Correlation between the IRF3 mRNA and HBsAg in positive HBeAg group. **(H)** Correlation between the IRF3 mRNA and TBA in positive HBeAg group. The correlation coefficiency (r) and the two-tailed *p*-values were then evaluated by Pearson’s test. *p-*values were considered to be significant when below 0.05.

We next evaluated the clinical correlations of INTS10 and IRF3 expression with key virological and metabolic parameters in HBeAg-positive patients. A significant negative correlation was observed between INTS10 levels and serum HBV DNA (r = −0.38, *p* = 0.003; Figure 3A), as well as with HBeAg (r = −0.41, *p* = 0.001; Figure 3B) and HBsAg levels (r = −0.27, *p* = 0.034; Figure 3C). Given the recognized impact of HBV infection on hepatic metabolic pathways, particularly bile acid metabolism, we also assessed TBA levels and found that INTS10 expression was inversely correlated with TBA (r = −0.26, *p* = 0.046; Figure 3D).

Similarly, IRF3 expression showed significant negative correlations with HBV DNA (r = −0.29, *p* = 0.026; Figure 3E), HBeAg (r = −0.28, *p* = 0.033; Figure 3F) and TBA (r = −0.26, *p* = 0.046; Figure 3H) in this patient group (all *p* < 0.05). However, no significant correlation was observed between IRF3 expression and the HBsAg levels (Figure 3G). Collectively, these findings suggest that both INTS10 and IRF3 are inversely associated with viral load, HBeAg levels, as well as TBA levels in HBeAg-positive CHB patients, implicating their potential role in modulating viral persistence infection.

### 3.5 Correlation between INTS10, IRF3 with HBV infection indicators in HBeAg-negative patients

**Figure 4:**
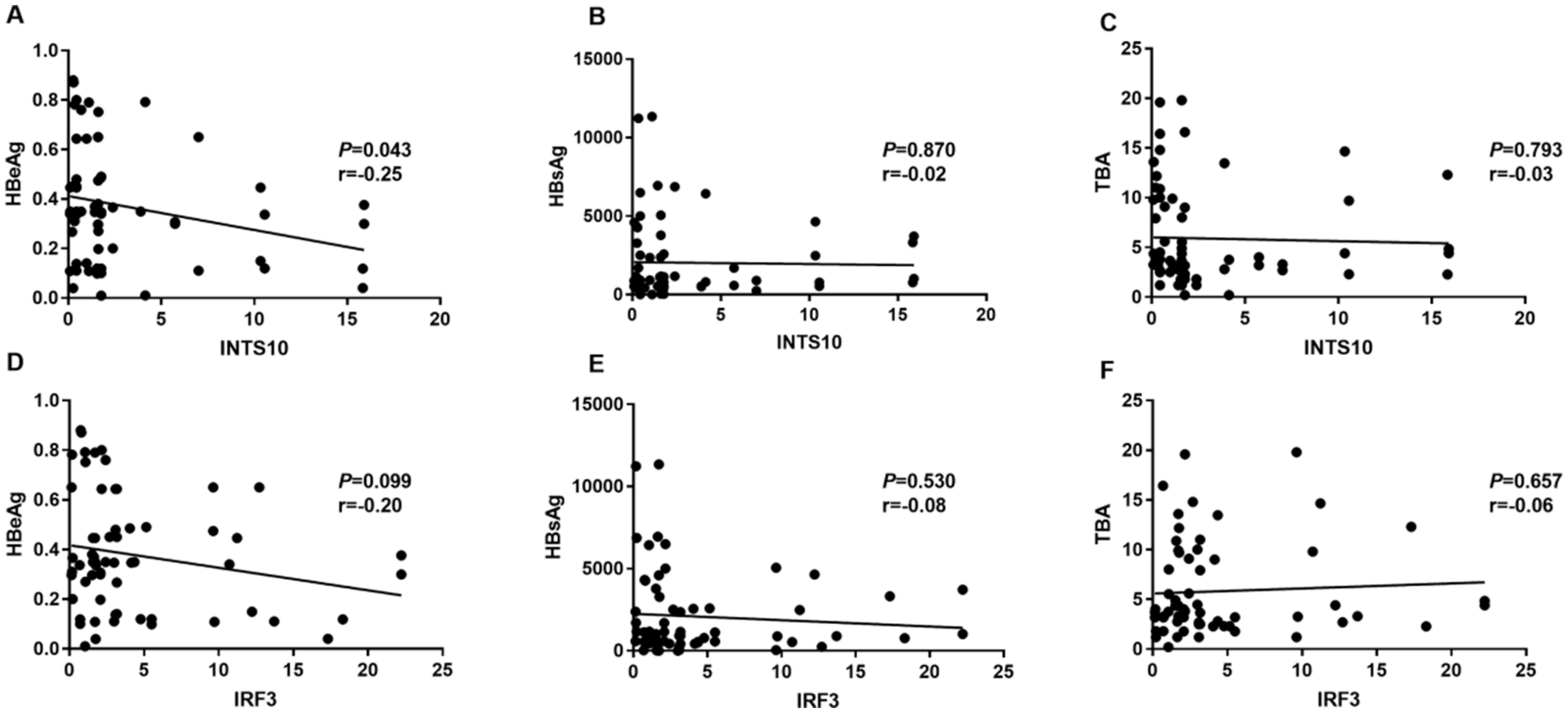
Correlation between the levels of INTS10, IRF3, HBeAg, HBsAg and TBA in HBeAg negative group. **(A)** Correlation between the INTS10 mRNA and HBeAg. **(B)** Correlation between the INTS10 mRNA and HBsAg. **(C)** Correlation between the INTS10 mRNA and TBA. **(D)** Correlation between the IRF3 mRNA and HBeAg. **(E)** Correlation between the IRF3 mRNA and HBsAg. **(F)** Correlation between the IRF3 mRNA and TBA. The correlation coefficiency (r) and the two-tailed *p*-values were then evaluated by Pearson’s test. *p*-values were considered to be significant when below 0.05.

Furthermore, serum INTS10 levels in HBeAg-negative patients exhibited a significant negative correlation with HBeAg (r = −0.25, *p* = 0.043; Figure 4A), but the relative expressions of INTS10 levels and HBsAg, TBA levels in HBeAg- negative CHB patients were no significant correlation (all *P* > 0.05; Figures 4B,C). Similarly, in our study, we did not found IRF3 levels and HBeAg, HBsAg, TBA in negative HBeAg CHB patients were in a significant correlation (all *P* > 0.05; Figures 4D–F). Taken together, these correlations were more likely to appear in the HBeAg-positive patients.

## 4 Discussion

Recent studies have identified INTS10 as a potential inhibitor of HBV replication. Notably, Zhou et al. demonstrated that INTS10 suppresses HBV replication in hepatocytes via the IRF3-mediated signaling pathway (Li et al., 2016; Barra et al., 2020). Consistent with these findings, our study revealed significantly decreased expression levels of both INTS10 and IRF3 in HBeAg-positive patients relative to their HBeAg-negative counterparts, along with a positive correlation between INTS10 and IRF3 expression. Furthermore, we identified a significant negative correlation between INTS10 levels and key clinical markers—including HBV DNA, HBeAg, HBsAg and TBA—specifically in HBeAg-positive patients. These results not only align with prior reports of reduced serum INTS10 and IRF3 in CHB patients, but also extend this understanding by delineating context-dependent associations with virological indicators.

A previous GWAS identified a novel locus within the INTS10 gene linked to susceptibility to HBV infection (Li et al., 2016). Additionally, some studies have demonstrated that reduced expression levels of INTS10 correlate with a poorer prognosis in cancer patients undergoing chemotherapy and the integrator can regulate the biogenesis of paraspeckles (PS), uncovering the associations among integrator, cancer biology, and chemosensitivity (Lee et al., 2021). Our results corroborate this by showing significantly higher INTS10 mRNA levels in HBeAg-negative patients than in HBeAg-positive patients. Moreover, specifically within the HBeAg-positive group, we observed a negative correlation between INTS10 expression and levels of HBeAg, HBsAg, HBV DNA, and TBA—a pattern absent in the HBeAg-negative group. This implies that active viral replication is associated with suppressed INTS10 expression. These findings underscore the pivotal role of INTS10 in regulating HBV replication and may have important implications for the prevention and management of HBV infection. In addition, studies suggest that INTS10 single nucleotide polymorphisms (SNPs) may interact with environmental factors such as tobacco smoking and alcohol consumption, collectively modulating the risk of HBV-related HCC. This implies the potential role of INTS10 SNPs in susceptibility to the disease progression of HBV infection (Wu et al., 2021).

Interestingly, a notable inverse correlation exists between the differential expression levels of INTS10 and NEAT1, a non-coding RNA implicated in the tumorigenesis and metastasis processes of HCC (Guo et al., 2015; Chen et al., 2025). Recent studies have indicated that NEAT1 is significantly overexpressed in prostate cancer, and its expression level is closely associated with both the malignancy of the tumor and the prognosis (Li et al., 2024). Based on this correlation, we hypothesize that patients with decreased INTS10 expression are likely to display elevated NEAT1 expression. This heightened NEAT1 activity may, in turn, exacerbate HBV replication, thereby accelerating disease progression and leading to less favorable clinical outcomes. Moreover, existing evidence has established a positive correlation between INTS10 and PinX1, a potent telomerase inhibitor. Notably, PinX1 expression is diminished in HBV-related HCC, a reduction that facilitates tumor growth and enhances tumorigenic potential (Huang et al., 2017; Sipila et al., 2025). Additionally, it has been observed that polymorphisms within the INTS10 gene exert synergistic effects on the transition from persistent HBV infection to HCC (Wu et al., 2021). Given these findings, future research endeavors should prioritize the elucidation of the underlying mechanisms through which INTS10 and its associated molecules modulate HBV progression and tumorigenesis.

The retinoic acid-inducible gene I-like receptor (RLR) pathway is of paramount importance in orchestrating the host immune response against HBV infection (Zeng et al., 2019; Lauterbach-Rivière et al., 2020). Wang et al. proposed that the HBV X protein (HBx) can impede virus-triggered activation of IRF3 and the subsequent induction of IFN-*β (*Wang et al., *2010**)*, while Zhou et al. provided compelling evidence that INTS10 can suppress HBV replication through an IRF3-dependent mechanism (Li et al., 2016). Furthermore, it has been observed that specifically targeting RIG-I and IRF3 can activate the RLR innate immune program. This activation is crucial for controlling HBV infection by suppressing covalently closed circular DNA (cccDNA), significantly reducing its half-life (t1/2) to days, in contrast to the weeks or months it persists without treatment (Barra et al., 2020). In alignment with these findings, our study revealed that the expression of IRF3 mRNA was notably higher in the HBeAg-negative group compared to the HBeAg-positive group. Additionally, we confirmed a positive correlation between the expression levels of INTS10 and IRF3, suggesting that INTS10 may facilitate enhanced clearance of HBV through the activation of IRF3. Consistent with this hypothesis, we identified a negative correlation between IRF3 expression and the levels of HBV DNA, HBeAg, and TBA in the HBeAg-positive group. This observation implies that the inhibition of viral replication mediated by IRF3 may be contingent upon the HBeAg status.

Emerging epidemiological evidence has illuminated a notable link between metabolic disturbances and HBV infection, underscoring the significant clinical associations that exist between HBV and host metabolic processes (Shlomai and Shaul, 2008; Shlomai et al., 2014). A number of studies have convincingly demonstrated that HBV infection can disrupt bile acid metabolism (Shlomai and Shaul, 2008; Li and Apte, 2015; Shi et al., 2016; Oehler et al., 2014). In our current investigation, we observed that serum TBA levels were markedly elevated in HBeAg-positive patients compared to those in HBeAg-negative patients. This finding aligns with the results reported by Xun et al (Xun et al., 2020). We hypothesize that this increase in serum TBA may be attributed to the involvement of Na + −taurocholate cotransporting polypeptide (NTCP), which serves as a liver receptor for HBV (Mao et al., 2019; Sun et al., 2024; Slijepcevic et al., 2017; Appelman et al., 2017). Specifically, HBV infection is likely to significantly alter hepatic bile acid uptake, consequently leading to an accumulation of serum TBA. Furthermore, our linear correlation analysis conducted in HBeAg-positive patients revealed a statistically significant negative correlation between INTS10 levels and serum TBA concentrations. These observations collectively suggest that metabolic pathways modified by HBV infection play a pivotal role not only in the pathogenesis of hepatitis B but also in the development of various metabolic disorders. Given these insights, further research is imperative to gain a deeper understanding of the complex metabolic and molecular pathways that are influenced by HBV, particularly those involving INTS10 and IRF3. Such investigations will be crucial for developing innovative therapeutic strategies aimed at achieving viral elimination and improving clinical outcomes for patients affected by HBV.

However, this study still has some limitations. Firstly, regarding the selection of research subjects, our focus was exclusively on patients with persistent HBV infection, while naturally recovered individuals were not incorporated into the study framework. To bolster the credibility and generalizability of the research conclusions, subsequent investigations ought to broaden the sample scope. This expansion should include inactive CHB patients, active CHB patients, and healthy individuals, thereby enabling a more thorough exploration of the expression characteristics of INTS10 across diverse populations. Secondly, as an observational study, the correlation results unearthed in this research necessitate verification through subsequent functional experiments. Although existing literature indicates that INTS10 may be involved in the anti-HBV immune response via the IRF3 pathway, the precise molecular mechanism by which it regulates HBV replication in hepatocytes remains elusive. Specifically, there is an urgent need to further clarify the interacting proteins associated with INTS10 and the functional network they form, so as to unveil its complete biological action pathway. Finally, this study is hampered by a relatively limited sample size, which may compromise the stability and representativeness of the research findings. Consequently, securing a larger sample size in future research holds paramount significance for addressing the aforementioned challenges and enhancing the overall quality of the study. By expanding the sample size, we can validate the existing discoveries with greater precision and gain deeper insights into the intricate action mechanism of INTS10 in HBV infection.

In summary, our research indicated a promising potential of INTS10 in inhibiting HBV replication and confirmed the significance of IRF3 in HBV infection progression, which could aid in the development of chronic infections. We observed elevated levels of INTS10 and IRF3 in patients who were HBeAg-negative. In particular, a negative relationship was identified between INTS10, HBeAg, HBsAg, HBV DNA, and TBA in patients with HBeAg-positive chronic hepatitis B, but not in those who were HBeAg-negative. These findings imply that HBeAg might influence the anti-replication roles associated with INTS10 and IRF3 in individuals affected by hepatitis B. Therefore, our results could enhance the clinical approach to managing HBV infections.

## Data Availability

The original contributions presented in the study are included in the article/supplementary material, further inquiries can be directed to the corresponding author.
